# Tuberculous Arthritis Diagnosed by Metagenomic Sequencing after Negative Microbiology Studies and a 4-Year Delay

**DOI:** 10.4269/ajtmh.26-0309

**Published:** 2026-08-11

**Authors:** Lu Yang, Li Xiang, Jin Rilong, Hu Yihe

**Affiliations:** Department of Orthopedics, First Affiliated Hospital, Zhejiang University School of Medicine, Hangzhou, China

## Abstract

Tuberculous arthritis of the knee is a rare form of extrapulmonary tuberculosis that often presents with nonspecific symptoms, leading to delayed or missed diagnosis, particularly in elderly patients with comorbidities. We report the case of a 70-year-old man with a 2-year history of left knee pain and recurrent swelling. The patient was under therapy for lung cancer. He underwent two arthroscopic procedures for both diagnostic and therapeutic purposes. Repeated standard tests for tuberculosis were negative before metagenomic next-generation sequencing (mNGS) identified *Mycobacterium tuberculosis* nearly 2 years later. After 1-year triple-antituberculous therapy, he remained asymptomatic at the 2-year follow-up. This case highlights a high index of suspicion for indolent infection in culture-negative chronic arthropathy. The use of mNGS—increasingly accessible and cost effective—enables rapid and sensitive diagnosis of paucibacillary extrapulmonary tuberculosis.

## INTRODUCTION

Tuberculous arthritis of the knee is a rare but clinically important form of extrapulmonary tuberculosis. Its clinical presentation is often insidious and nonspecific, particularly in elderly and immunocompromised patients, leading to frequent misdiagnosis as degenerative joint disease or other inflammatory arthropathies. The paucibacillary nature of the infection further complicates diagnosis as conventional microbiological methods, including smear microscopy, culture, and nucleic acid amplification tests, frequently yield false-negative results, resulting in prolonged diagnostic delays and progressive joint destruction.

## CASE REPORT

A 70-year-old man presented with a 2-year history of left knee pain. He had no history of trauma or preceding infection. The pain was intermittent, accompanied by recurrent swelling, and exacerbated by prolonged walking. It was nonradiating, and there was no locking or clicking. His medications included occasional analgesics and the use of traditional herbal plasters.

The patient worked as a miner for 1 year and had a history of silicosis. He had been diagnosed with small cell lung cancer 1 year before presentation. The patient had completed five cycles of dose-reduced Etoposide + Carboplatin (EC) chemotherapy and one course of stereotactic body radiation therapy (50 Gy in five fractions). He did not receive immunotherapy owing to compromised lung function and the associated risk of immune-related pneumonitis. On examination, he was afebrile with stable vital signs. Cardiopulmonary and abdominal examinations were unremarkable. The left knee demonstrated a mild effusion with preserved range of motion; there was no crepitus, joint-line tenderness, palpable osteophytes, warmth, or erythema. Radiographs of the left knee revealed Kellgren–Lawrence Grade 3 degenerative changes involving both the medial and lateral compartments.

The patient was initially managed with physical therapy and etoricoxib. He returned 2 months later with minimal symptomatic improvement. He remained afebrile and denied weight loss. There was no involvement of other joints or systemic symptoms. Laboratory studies revealed a normal C-reactive protein level. The white cell count was 6.92 × 10^9^/L, with a neutrophil proportion of 72.8% and relative lymphopenia of 15.8%. The erythrocyte sedimentation rate was 23 mm/hour. Testing for antinuclear antibodies, antineutrophil cytoplasmic antibodies, serum immunoglobulins, and protein electrophoresis was within normal limits.

**Figure 1.**
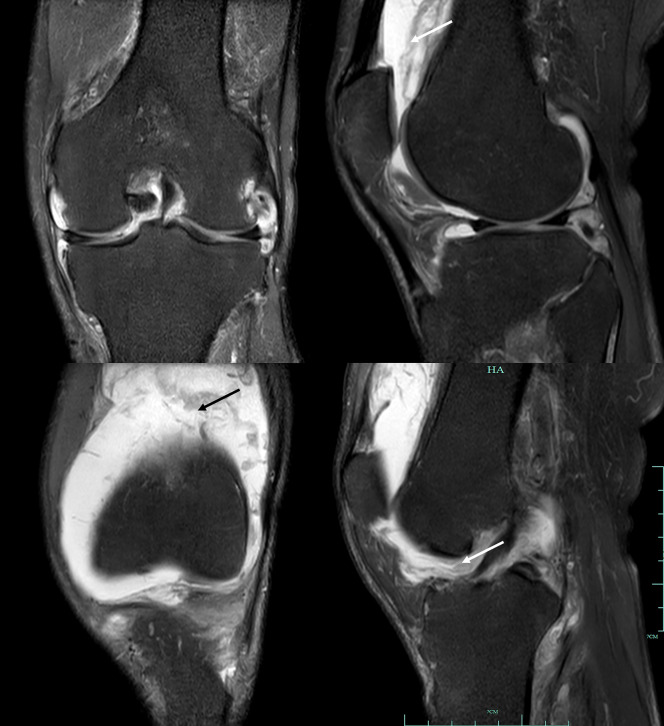
Magnetic resonance images of the patient. White and black arrows indicate cloud-like material within the joint cavity.

Magnetic resonance imaging (MRI) revealed degenerative changes of the knee, with marked synovial hyperplasia and a moderate-to-large joint effusion (Figure 1). The radiology report noted that pigmented villonodular synovitis (PVNS) or infection should be considered in the differential. Arthrocentesis yielded clear, yellow-tinged synovial fluid. Analysis showed a white blood cell count of 3,200/cm^3^, lactate dehydrogenase of 289 U/L, protein of 43.3 g/L, glucose of 4.0 mmol/L, and adenosine deaminase of 28 U/L. The smear and cultures were negative.

**Figure 2.**
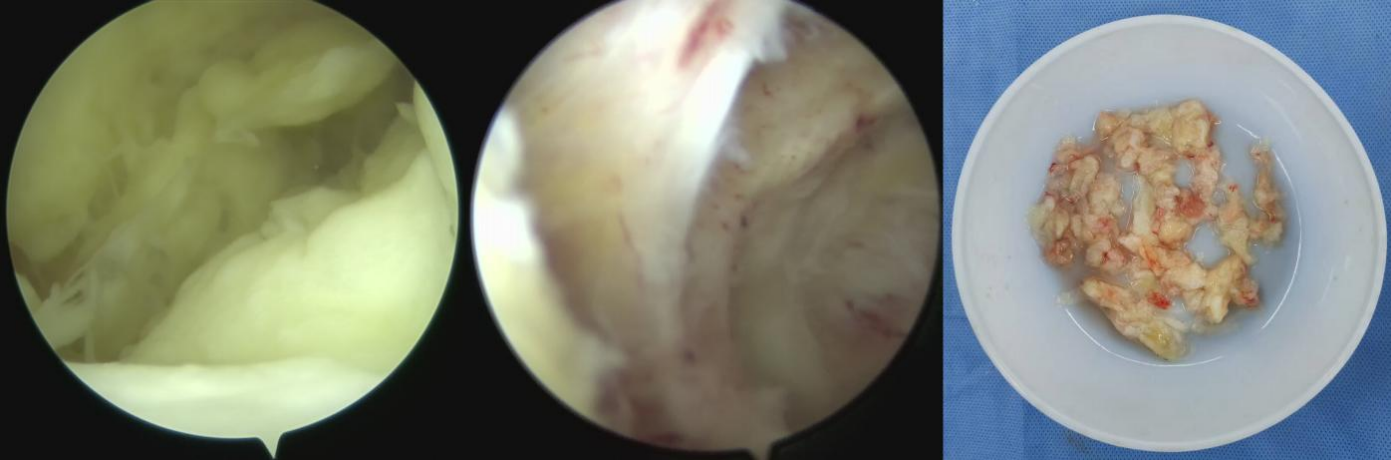
Arthroscopic views before (left panel) and after (center panel) debridement. Gross view of the cloud-like material (right panel).

One month later, the patient underwent knee arthroscopy. The procedure confirmed degenerative changes involving the meniscus and cartilage. However, the joint cavity was unexpectedly found to contain copious, soft, whitish, glistening material of irregular morphology. Most of this material adhered to the synovium but could be completely detached with minimal traction; a minor portion was free floating (Figure 2). Aerobic and anaerobic bacterial cultures; Gram stain; tuberculosis mycobacteria growth indicator tube cultures; and acid-fast bacillus smear of joint fluid, the white cloud-like material, and synovium were all negative. Histopathology revealed fibrinous exudation and scattered inflammatory cell infiltration; no acid-fast bacilli were identified. An empirical 4-week course of moxifloxacin was initiated postoperatively.

Despite the initial arthroscopy and empiric antibiotic therapy, the patient’s knee pain and swelling recurred after 6 months. He underwent several arthrocentesis procedures over the following months, with synovial fluid ranging from clear to sanguineous on repeated occasions and negative bacteriological examination. Intra-articular injections of hyaluronic acid, compound betamethasone, and ropivacaine provided only transient symptomatic relief. Sixteen months after the initial arthroscopy, a repeat MRI demonstrated persistent synovitis and effusion, similar to the preoperative findings. The presence of sanguineous synovial fluid raised suspicion for PVNS. The posterior-medial and posterior-lateral portals were absent, which means that the posterior compartments may not have been adequately debrided during the initial arthroscopy—a recognized cause of recurrence after surgical treatment of PVNS.^1^ A second arthroscopic surgery was undertaken to achieve more thorough debridement and to obtain representative synovial tissue from multiple sites, including the posterior compartments, to maximize the diagnostic yield of microbiologic and histopathologic studies.

**Figure 3.**
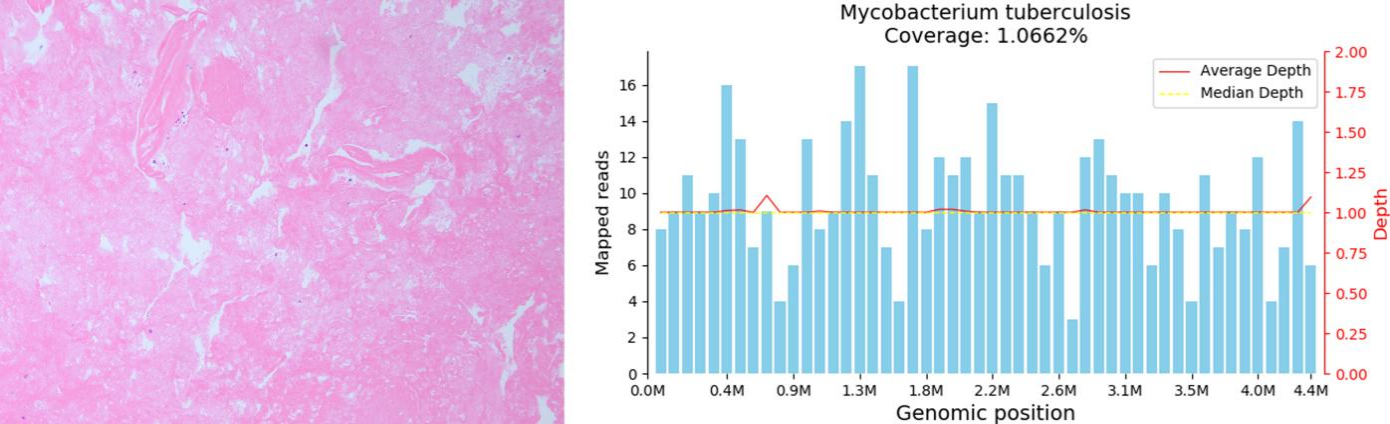
Hematoxylin and eosin-stained section (×100; left panel) and bar graph of the metagenomic next-generation sequencing result (right panel). The red line indicates average depth; the yellow dashed line indicates median depth. M, million.

The intraoperative findings were similar to those of the initial surgery. All five mycobacterial smears and cultures from three arthrocenteses and two arthroscopic specimens together with two histopathological acid-fast stains from two arthroscopic specimens were negative. Metagenomic next-generation sequencing (mNGS) of synovium, however, identified 1,064 specific reads of *Mycobacterium tuberculosis* (Figure 3). The patient received a 12-month course of triple-drug antituberculous therapy consisting of isoniazid (5 mg/kg per day), rifampicin (10 mg/kg per day), and ethambutol (15 mg/kg per day). The regimen was administered in two phases: 1) an intensive phase of 2 months with all three drugs isoniazid, rifampicin, and ethambutol (HRE) followed by 2) a continuation phase of 10 months with isoniazid and rifampicin (HR) for a total duration of 12 months. No pyrazinamide was included because of concerns regarding hepatotoxicity in a patient with recent chemoradiation. He remained asymptomatic during the 2 years of follow-up. At last follow-up, the patient had a “forgotten knee” with full range of motion.

## DISCUSSION

Knee pain accounts for approximately 5% of all primary care visits in adults. Osteoarthritis is the leading cause of chronic knee pain in patients older than 45 years of age. In early-stage osteoarthritis, pain is typically intermittent and related to weight-bearing activity. As in the current case, diagnosis is primarily based on history and physical examination. Diagnosis of osteoarthritis using the criteria of chronic knee pain and at least three of the criteria of age older than 50 years old, <30 minutes of morning stiffness, crepitus with motion, bony tenderness, bony enlargement, and no palpable synovial warmth has a sensitivity of 95% and a specificity of 69%. The correlation of knee symptoms with radiographic findings is only poor to moderate.^2^ Therefore, radiographic study is not routinely required, and it is generally reserved for differential diagnosis, severity assessment, or surgical planning. Integrating clinical assessment with selective radiographic and laboratory testing slightly reduces sensitivity by 3% to 4%, but it significantly improves specificity by 6% to 17%.^3^

Besides degenerative osteoarthritis, chronic knee pain may also arise from inflammatory arthritis (e.g., rheumatoid or psoriatic arthritis), crystal arthropathy (e.g., gout or pseudogout), proliferative disorders (e.g., PVNS or synovial chondromatosis), and indolent infectious arthritis. Indicators of high risk are pain at rest, polyarthralgia, persistent warmth, palpable effusions, and lack of response to therapy. Diagnostic complexity increases in patients with significant comorbidities, such as prior malignancy, infection, or exposure to immunosuppressive therapies. This patient’s history of silicosis, lung cancer, and recent chemoradiation broadened the differential to include metastatic disease, treatment-related reactive arthritis, and atypical infections. Notably, silicosis is an independent risk factor for tuberculosis,^4^ which was somehow overlooked in this case. Furthermore, patients with lung cancers had a 9-fold-higher rate of developing active tuberculosis compared with those without cancer.^5^

Tuberculous arthritis, although uncommon, represents an important diagnostic consideration in patients presenting with monoarticular pain and swelling, particularly in regions with a high burden of tuberculosis or in individuals with immunosuppressive conditions. Osteoarticular tuberculosis accounts for 1% to 3% of all tuberculosis cases. The knee joint is involved in 8% to 10% of all skeletal tuberculosis, ranking second after spine and hip involvement.^6^ Musculoskeletal tuberculosis typically involves a single joint or vertebral segment, although multifocal disease occurs in approximately 10% of cases. Clinical presentation varies by age. In children, knee swelling or a slight limp is often the first noticed symptom because of synovial involvement. In adults, particularly the elderly, weight-bearing knee pain is a prominent feature because of earlier invasion of the articular cartilage. Constitutional symptoms, such as fever, fatigue, anorexia, weight loss, night sweats and anemia, are frequently absent in tuberculous knee arthritis, further obscuring the diagnosis. As illustrated in the present case, tuberculous arthritis in immunocompromised or elderly patients may follow an indolent course with chronic pain, swelling, and gradual functional decline, easily mimicking osteoarthritis or other noninfectious inflammatory conditions.

Radiographs may show periarticular osteopenia and joint-space narrowing in late stages of tuberculous arthritis but remain normal before joint structures are extensively damaged. In early stages, MRI can reveal synovitis, joint effusion, cartilage damage, and bone marrow lesions. In the current case, the substantial effusion and synovitis observed on imaging raised suspicion of an underlying etiology. However, the white cloud-like material was misinterpreted as synovitis on MRI, leading to consideration of proliferative disorders, including PVNS and synovial chondromatosis.

Diagnosing tuberculous arthritis remains challenging given the paucibacillary nature of the infection and the limited sensitivity of conventional microbiological methods. Synovial fluid in tuberculous arthritis typically appears purulent, with reduced glucose and a white cell count of 15,000 to 20,000/cm^3^. An adenosine deaminase level exceeding 40 to 50 U/L in synovial fluid raises strong suspicion of tuberculous infection.^7^ Notably, this patient presented with atypical findings; the synovial fluid was clear or sanguineous rather than purulent, and laboratory studies revealed only mildly elevated synovial fluid leukocyte count, synovial fluid adenosine deaminase level, and serum inflammatory markers.

Definitive diagnosis of tuberculosis arthritis requires identification of *M. tuberculosis* in synovial fluid or tissue by culture, nucleic acid amplification tests, or histopathological examination. Immunologic assays as well as initial smears and cultures frequently yield negative results. In such instances, confirmation may be pursued through repeated, more invasive microbiological sampling and biopsy for histopathologic analysis. Alternatively, when clinical and radiographic suspicion is strong, a diagnostic trial of empiric antituberculous therapy may be warranted. The decision must weigh a high pretest probability of untreated disease against the risks of prolonged, potentially unnecessary multidrug therapy and its associated adverse effects.

The even distribution pattern of the mNGS result indicates that all aligned reads were specifically matched to *M. tuberculosis* genomic loci rather than clustered in a single region—a pattern that would raise concern for contamination or nonspecific alignment. This finding in conjunction with the clinical presentation of chronic monoarticular arthritis, synovial histopathology demonstrating with cellulose exudation, and successful treatment established the diagnosis of tuberculous arthritis. One of the challenges in diagnosing tuberculosis lies in the frequently low bacterial load in patient specimens, making the selection of methods with the lowest limit of detection (LoD) critical for improving diagnostic yield. The LoD varies substantially across technical platforms; conventional Xpert mycobacterium tuberculosis/Rifampicin has an LoD of approximately 113 colony-forming unit/mL, whereas the upgraded Xpert Ultra reduces this to approximately 16 CFU/mL (and even 0.16 CFU/mL in cerebrospinal fluid). Novel targeted next-generation sequencing can achieve an LoD as low as 10 copies/mL, demonstrating superior sensitivity in low-burden samples; in contrast, although mNGS is limited by low *M. tuberculosis* sequence counts and host nucleic acid interference, it retains unique complementary value in difficult-to-diagnose and mixed-infection cases through optimized interpretation thresholds. Therefore, for suspected tuberculosis with low bacterial burden, methods with lower LoD are recommended as the priority to enhance diagnostic positivity.^8^^–^^10^ In China, the cost of this technology has fallen below $400, and results can be available within 48 hours, making it an increasingly accessible tool in complex diagnostic scenarios.

Treatment of tuberculous arthritis requires a prolonged course of multidrug antituberculous therapy, with surgical intervention reserved for cases of advanced disease, substantial joint destruction, or inadequate response to medical management. Although debridement may improve drug penetration and reduce antigenic burden, the optimal timing and extent of surgical intervention remain subjects of ongoing debate.^6^^,^^11^^,^^12^ In the current case, the gross morphology of the joint contents along with their easy separation from the synovium argues against synovial hyperplasia and favors a depositional process. We believe that these substances represent a specific manifestation of chronic inflammation. Further study is needed to elucidate the nature of this underreported cloud-like material. Whether the infection could have been eradicated without surgically addressing the observed intra-articular deposits—or whether this material would have resolved with adequate antituberculous therapy alone—remains an open question.

This case illustrates several factors that contribute to the delayed diagnosis of tuberculous arthritis. First, the patient’s advanced age and protracted clinical course initially directed attention toward degenerative joint disease, a common pitfall in elderly populations. Second, his comorbid lung cancer and prior chemoradiation raised alternative considerations, such as metastatic disease or treatment-related synovitis, further diverting attention from infectious causes. Third, the absence of constitutional symptoms, atypical synovial fluid, and only mild elevation of inflammatory markers obscured the underlying tuberculous infection. Finally, repeated negative results from conventional tuberculosis tests highlight the diagnostic challenges inherent in paucibacillary extrapulmonary tuberculosis. Taken together, these factors underscore the importance of maintaining a high index of suspicion for atypical chronic infection, even when initial workup is unrevealing but the clinical picture overlaps with more common conditions. In culture-negative chronic arthropathy, timely incorporation of advanced molecular diagnostic techniques may help avert delays of diagnosis.
